# NMRFx Processor: a cross-platform NMR data processing program

**DOI:** 10.1007/s10858-016-0049-6

**Published:** 2016-07-25

**Authors:** Michael Norris, Bayard Fetler, Jan Marchant, Bruce A. Johnson

**Affiliations:** 1 One Moon Scientific, Inc., 839 Grant Ave., Westfield, NJ 07090 USA; 2 Howard Hughes Medical Institute, University of Maryland Baltimore County, 1000 Hilltop Circle, Baltimore, MD 21250 USA; 3 Department of Chemistry and Biochemistry, University of Maryland Baltimore County, 1000 Hilltop Circle, Baltimore, MD 21250 USA; 4 Structural Biology Initiative, CUNY Advanced Science Research Center, 85 St. Nicholas Terrace, New York, NY 10031 USA

**Keywords:** NMR, Data processing, Signal processing, Non-uniform sampling

## Abstract

NMRFx Processor is a new program for the processing of NMR data. Written in the Java programming language, NMRFx Processor is a cross-platform application and runs on Linux, Mac OS X and Windows operating systems. The application can be run in both a graphical user interface (GUI) mode and from the command line. Processing scripts are written in the Python programming language and executed so that the low-level Java commands are automatically run in parallel on computers with multiple cores or CPUs. Processing scripts can be generated automatically from the parameters of NMR experiments or interactively constructed in the GUI. A wide variety of processing operations are provided, including methods for processing of non-uniformly sampled datasets using iterative soft thresholding. The interactive GUI also enables the use of the program as an educational tool for teaching basic and advanced techniques in NMR data analysis.

**Electronic supplementary material:**

The online version of this article (doi:10.1007/s10858-016-0049-6) contains supplementary material, which is available to authorized users.

## Introduction

**Fig. 1:**
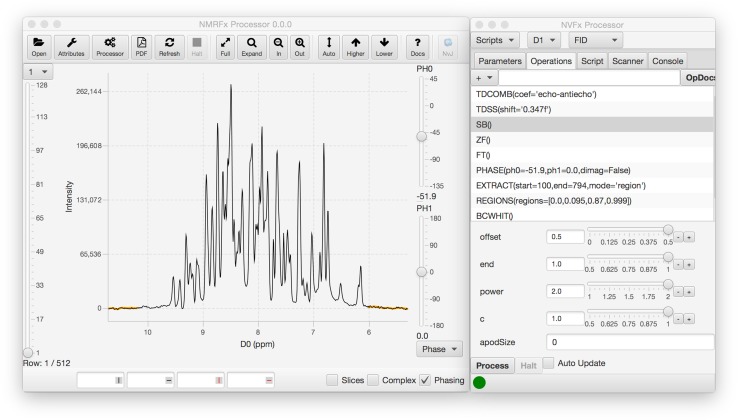
Screenshot of NMRFx graphical user interface. The main window is shown with the first processed first row of an HSQC spectrum. The smaller window shows the list of operations that are currently specified for processing of the first dimension. Selecting an operation in the list populates the area below with controls for adjusting parameters relevant to that operation. Inserting, moving or deleting operations or changing parameters immediately updates the displayed vector with application of the new processing scheme

Any use of NMR data requires that the raw data be transformed using a variety of signal processing algorithms (Hoch and Stern 1996). Given the relatively low sensitivity of the NMR experiment, the vast variety of experimental techniques for magnetization transfer, and the expense of preparing many samples, proper data analysis is crucial to ensuring that the information contained in the raw data can be effectively transformed into knowledge about the molecular system under study. A variety of non instrument vendor choices for users exist in visualization (Bartels et al. 1995; Johnson 2004; Johnson and Blevins 1994; Keller; Lee et al. 2015; Vranken et al. 2005), but fewer actively supported ones exist for data processing (Delaglio et al. 1995; Hoch and Stern 1996). In particular, we’re not aware of an open source, cross-platform, comprehensive processing program with a modern graphical interface. In this manuscript we describe a new program for processing NMR data. The application builds on some low level code from NMRViewJ (Johnson 2004; Johnson and Blevins 1994), adds new processing operations that include support for non-uniformly sampled data, and adds a totally new graphical user interface (GUI) for setting up the processing (Fig. 1). The combination of some legacy code from NMRViewJ with the use of the new Java GUI toolkit, JavaFX, led to the choice of the name, NMRFx Processor.

As noted in the paper introducing nmrPipe (Delaglio et al. 1995), users of NMR data are often not experts in data processing or even NMR theory. Accordingly it’s vital that processing tools be readily usable by non-experts. To facilitate this, most datasets can be processed in NMRFx Processor with sensible default operations and parameters with minimal user input. At the same time, we consider it important that NMRFx provide insight into the processing operations and that use of NMRFx has educational value. To this end, any addition of new processing operations or modification of parameters by the user is applied on the fly, clearly demonstrating their effect. This not only makes it more likely that users will maximize the information content available from their data, but also serves as a useful educational tool for teaching basic and advanced techniques in NMR data analysis.

NMRFx Processor provides a new option for processing NMR datasets and includes both command line and GUI based processing. This cross-platform application runs on Windows, Mac OS X, and Linux. The inclusion of many processing options, including protocols for non-uniformly sampled data, allow its application to a wide variety of one dimensional and multi-dimensional NMR datasets.

## Methods

### Supported platforms

Users differ in their preference for type of computer for daily use. Various versions of Mac OS X, Windows, and Linux, some in both 32 bit and 64 bit versions, are widely used. Providing software tools that work across all of these platforms is a challenge to which three strategies are generally applied. First, native applications can be specifically developed for each platform. Second, applications for a single platform (typically Linux) can be developed and then run in a virtual operating system (for example, VMWare Fusion, Parallels, VirtualBox) on the user’s preferred platform. Third, applications can be developed in a cross-platform environment like Java so that the same application code can be run on any of the platforms. For several reasons, we’ve chosen the latter approach. It allows more developer time to be spent on application development, rather than getting the software to compile and run natively on diverse computers. Unlike the use of virtual operating system environments, it allows execution with essentially native appearance and integration, and doesn’t require partitioning processors and memory between the native and virtual operating system. Java also provides rich features for harnessing the power of multi-core computers, access to extensive libraries of cross-platform tools, and features of a modern object-oriented language.

### Software tools

NMRFx and its associated data engine are written in a combination of Java and Python. Java code is used for all low-level processing and display code. The current release of the code is compiled with Java version 1.8. In the normal distribution of NMRFx the Java Runtime Environment is included in the installer and installed along with program code. This removes the necessity of ensuring a compatible version of Java is installed on their computer, essentially making the use of Java transparent to the end-user.

The GUI of NMRFx (Fig. 1) is created using JavaFX, a recently developed GUI toolkit which is standard with Java version 1.8 and later. The actual graphical displays are created in a combination of FXML (an XML based format for defining JavaFX interfaces) and Java code.

The Python programming language is used for processing scripts. Python as used in NMRFx is actually implemented with Jython (http://www.jython.org), which is a Java based implementation of Python. Jython 2.7.0 is used in the current release and is essentially feature complete with C Python 2.7.

Building of NMRFx is managed with Maven and source code is stored in the Mercurial source code management system. Unit tests are developed with JUnit and Python unittest.

The source code for NMRFx Processor (for both the GUI and low-level processing) will be available under the GNU General Public License (GPL) version 3.

### Processing engine

**Fig. 2:**
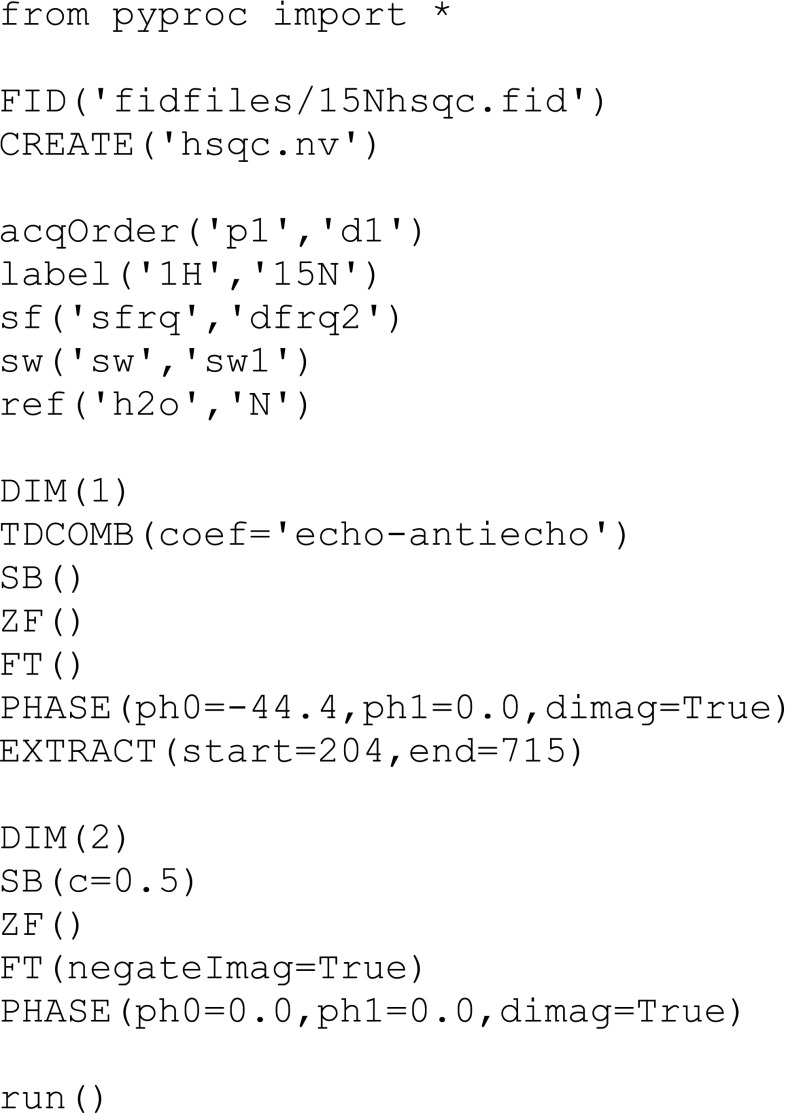
Example of an NMRFx Processing Script. This script is used to process a 2D HSQC dataset. It includes reference commands and processes each dimension with sine-bell apodization, zero-filling, Fourier transform and phasing. The first dimension has an EXTRACT command to extract the signal-containing region

A hallmark of NMR is the extensive variety of experimental techniques implemented in pulse programs. The configurable options and parameters exposed in the NMRFx GUI are designed to be sufficiently flexible to support this wide variety. However, in many cases the use of user-defined scripts will be the most powerful and appropriate option. Accordingly, the NMRFx processing engine uses scripts that are expressed in the Python programming language (Fig. 2).

These scripts consist of three main sections. File operations (FID, CREATE) specify the raw FID(s) to open and the dataset(s) to create. At present, NMRFx Processor can read Agilent (Varian), Bruker, and 1D JCAMP files and it stores the resulting spectrum data in the sub-matrix dataset format used by both NMRViewJ (Johnson and Blevins 1994) and Sparky (Lee et al. 2015) These two formats differ only in the information stored in the header and the output choice is determined by the specified file name extension (.nv or.ucsf). Converters for outputting the data in formats used by other NMR visualization programs are under development and the NMRViewJ file format is documented in the online manual so that other developers can add their own file readers and converters. Referencing commands (sw, label etc.) specify parameters used in dataset referencing (see below) and data sizes. Processing commands specify the actual signal processing operations to be performed and their use will be described here.

Processing commands are added by first specifying a DIM command (with an argument specifying the dataset dimension (1, 2, …). Subsequent commands, that refer to processing operations (ZF, SB, FT, etc.), append the specified operation on to a list of operations that will be performed on that dimension. At present, over 60 different processing commands are available. The most commonly used are listed in Table 1, and the complete list is in Online Resource 1. These processing operations, as used in the script, correspond to methods defined in the Python programming language and standard use of method arguments apply. Most commands have optional arguments with default values. Arguments can be specified with their argument names, **EXTRACT(start** **=** **100,end** **=** **500)**, or without argument names, **EXTRACT(100,500)**, if they are provided in the order they appear in the method definition. All operations have at least brief documentation embedded in the method definition and these are extracted to generate overall documentation for all available operations. All scripts must end with a **run** command. Commands in the script prior to the **run** command define what processing will occur. The **run** command itself triggers execution of the low-level processing code that carries out the specified processing operations.

**Table 1:** Common processing operations

Operation	Description
AUTOPHASE	Automatic phasing of current vector
BCPOLY	Baseline correction with polynomial
BCWHIT	Baseline correction with smoothed line
CSHIFT	Circular shift
DC	Baseline correction by linear offset
EXPD	Apodization with exponential decay
EXTRACT	Extract a range of the spectrum (for example the left half of an amide detected spectrum)
FDSS	Frequency domain solvent suppression
FT	Fourier transform
GM	Apodization with Lorentz to Gauss transform
HFT	Hilbert tranform to recover imaginary components of real valued spectrum
IST	Iterative soft thresholding of a 1D vector (used for 2D datasets)
ISTMATRIX	Iterative soft thresholding of an nD matrix (used for 3D and higher datasets)
LP	Extend the vector with linear prediction
LPR	Replace starting points with linear prediction
MAG	Magnitude calculation
PHASE	Adjust the phase of the spectrum
POWER	Power calculation
REGIONS	Specify regions of the vector. Typically used prior to baseline correction.
REVERSE	Reverse the spectrum
SB	Apodization with a sine or cosine window
SCRIPT	Execute a Python script. The current vector is accessible as a Python object named “vec”
TDCOMB	Form linear combinations of time domain signal using specified coefficients
TDSS	Time domain solvent suppression
ZF	Zero fill

Complete list in Online Resource 1

**Fig. 3:**
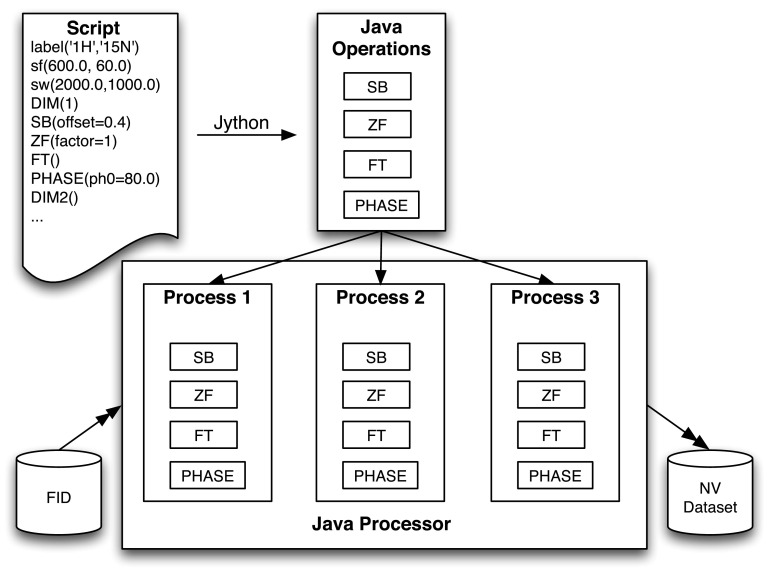
Flow chart of the NMRFx processing scheme. The Python script is interpreted by Jython (the Java version of Python that is embedded in NMRFx). This generates a set of Java operations, one for each processing command. The Java Processor in NMRFx replicates the list of Processes so that the processing can take advantage of multiple CPUs or CPU cores. The Processor also manages the reading and writing of data values

A brief description of the implementation will aid in understanding the relation of the Python processing operation commands, and the actual processing (Fig. 3). The DIM command creates a new instance of a Java object corresponding to the Process class. The subsequent Python processing operation commands don’t normally perform actual processing, instead they append an instance of a Java object corresponding to that operation on to a list maintained by the Process object. Thus, at the end of the script in Fig. 2, two Process objects will exist. The first contains a list of the six operations (each defined by a Java object that implements the code for the operation) used in processing the first dataset dimension, and the second contains a list of the four operations used in processing the second dataset dimension.

The **run** command at the end of the script triggers a Processor object to load vectors from the raw dataset and make them available to the Process object for the first dimension. The Process object requests a set of vectors from the Processor and then steps through the series of operations and calls the method in each operation to process the current list of vectors. This is repeated until all available vectors are processed. The operation list for each Process object has an implicit operation appended that writes the processed vectors out to the destination dataset. The overall scheme is repeated for each available Process object, typically one per dimension. After the second dimension, vectors to be processed are read, not from the original FID file, but from the destination so that the processing is done in place on a single final file. Processing is complete when all Process objects have been executed.

### Parallel computation

The time required for dataset processing is an important concern in NMR processing. Rapid processing allows the user to try, possibly interactively, multiple processing schemes and parameter values so that the final spectral quality can be optimized. Furthermore, many newer experimental protocols involving non-uniform sampling can save considerable time in experimental acquisition, but may require complex and lengthy processing schemes. One way to decrease the time required for dataset processing is to perform calculations in parallel. This is possible because almost all modern computers, even simple laptops, use processors that have multiple cores that can operate in parallel. More expensive desktop and server computers may include multiple CPU chips, each with multiple cores. Utilizing these multiple cores, however, is not generally automatic and requires the processing software to be specifically designed to use them.

The NMRFx processing engine has been engineered from the very beginning to take advantage of multiple core computers. Parallelizing the processing scheme is in principle straightforward since most NMR processing involves applying the same set of operations to a large number of subsets of the data. These data subsets may be the multiple 1D vectors parallel to a given dataset dimension involved in standard processing schemes, or higher dimensional matrices used in schemes for processing non-uniformly sampled data.

As noted, NMR data is readily amenable to parallel processing, but in practice this requires careful software development. The Processor object mentioned above can execute the processing operations in parallel. When the processing script’s **run** command is called the Processor checks for the number of operations to run in parallel. Prior to triggering each Process object to execute its operations, the Processor clones the Process object for each parallel operation. Actual parallel operation is done using Java’s code for setting up a Thread pool with one thread for each planned parallel operation. Each of the cloned Process objects is submitted to the Thread pool. Two parameters control how the parallelization works: the number of threads to be created (and thereby processing operations to run in parallel), and the number of vectors that each Process should request at a time. The thread number defaults to half the value returned by the Java Runtime.availableProcessors() method. This typically yields a number equal to the number of processor cores. The raw number from Runtime.availableProcessors is generally twice the number of processor cores on Intel Processors as Intel’s Hyper-Threading technology (Marr et al. 2002) makes each core appear to the operating system as two processors. The number of vectors per process will control the amount of memory used during processing. Too many vectors per process may result in the application exceeding available memory, while too few may increase the proportion of overhead time used by each process. The number of vectors requested at a single time by a Process object is by default set to the total number of vectors to be processed divided by the number of processors, or 64, whichever is smaller. The user can modify both the thread number and vector number. The key feature of this parallelization is that it requires no extra effort, such as script modification, by the user. Parallel processing happens automatically and transparently to the user.

### Graphical user interface

NMRFx Processor provides a full GUI for viewing NMR data in both the raw FID and processed spectrum and in interactively defining processing scripts. Figure 1 shows a screenshot of the GUI in action. The main display window is capable of displaying both raw FIDs and processed spectra. The code for spectral display, which is derived from NMRViewJ code, is capable of basic 1D vector and 2D contour plots of data of any dimension number. Clicking a button in the toolbar will open NMRViewJ and display the current dataset so as to allow more extensive visualization and analysis. The main window contains a toolbar with options for standard display control (vertical height, zooming etc.). A spectrum attributes window, similar to that in NMRViewJ, can be displayed to control colors, view orientation and slice display parameters.

The separate Processor window has features for interactively specifying processing parameters and operations. Five tabbed display regions provide access to Parameters (labels, spectrometer frequency etc.), Operations (processing commands), Scripts (the Python processing script), the Scanner tool (used for batch processing of multiple datasets) and a Console. The Operations tab provides access to a menu of processing operations, grouped by categories like Apodization, Phasing, and Transforms. Choosing an operation from the menu inserts the operation (with default parameters) into the processing list. Operations have a preferred ordering so, for example, a Phase operation will be inserted after a Fourier Transform operation, and an apodization operation will be inserted before the Fourier Transform command. As the default order may not be appropriate, it is possible to click and drag operations to new positions in the list.

Most operations have associated optional parameters, which are specified in scripts as arguments to the Python command. These parameters can be interactively adjusted by controls in the GUI. Selecting an operation in the operation list will display the corresponding controls in the area below the list.

Any changes in the processing operations affected by adding, deleting, or moving operations, or changing the parameters associated with an operation, will result in the application of the operations to the current FID vector (or vectors if the processing requires combinations of vectors). The processed vector displayed in the data display region will immediately be updated. This allows the user to interactively change the processing scheme and observe the effects on the FID. This display update works with both the directly detected FIDs and, in the current version, with indirectly detected FIDs from 2D datasets. Display of the indirect FID (obtained by locating correct data points from the raw FID file) allows visual analysis of the processing scheme for the indirect dimension (in 2D datasets) prior to doing the transform of the direct dimension. We expect to extend this capability to higher dimensional spectra, but this is not a priority as the lower signal to noise can preclude useful analysis of a single processed indirect FID in higher dimensional spectra.

Some processing schemes may require various linear combinations of FIDS such as forming a new pair of FIDs from the addition and subtraction of the original pair. NMRFx Processor allows the user to select and display each member of a set of vectors to be combined. The interactive processing can include the linear combination operation so the user can assess whether the correct combination has been made.

When using multi-dimensional datasets, processing of the complete dataset (or specified subset) is initiated by clicking the “Process Dataset” button. Upon completion of processing, the resulting spectrum file will be opened and displayed as a 2D contour plot. For smaller datasets, where the processing can be done rapidly (under 1 s) it is feasible to trigger processing and dataset display interactively while adjusting operations and parameters. Selecting the “Auto Update” button activates this interactive mode. We expect that an NMRFx feature under development, where the dataset is kept fully in memory, will make this feasible for larger datasets.

The Script tab is used to view, edit, open and save Python processing scripts. Selecting the Script tab will immediately update the displayed script based on selected parameters and operations. The script can be manually edited prior to clicking the “Process Dataset” button. The edited script will be used for the processing. In the current version, leaving the script tab and returning will reconstruct the script from the operation list, losing any manual changes. When the dataset is processed, the current script will be saved as a file named “process.py” in the same directory as the FID file. Menu commands can be used to save or load the processing script to or from a specified file. When a script is loaded it is parsed and the parameters and operation lists for all dimensions are updated based on the script. Script commands for selecting the dataset and output file (FID and CREATE) are replaced with commands based on the currently active dataset. This allows scripts setup with one dataset to be loaded and used to process different (but compatible) datasets. Arbitrary Python code that is added to the script outside of NMRFx will not be included.

Hardcopy output is available in PDF format for 1D vector and 2D contour displays. Exporters for other formats such as PNG and SVG are planned.

### Batch processing

Many applications of NMR involve collecting multiple datasets with the same pulse sequence and parameters. NMRFx Processor provides support for batch analysis both through the GUI and through Python scripting.

The Scanner tab provides tools for batch processing of multiple datasets. This is particularly useful for applications like relaxation analysis, ligand titrations, metabolomics and fragment screening where multiple datasets are processed with identical operations. All compatible NMR datasets within a selected directory will be found and presented in a list. Any of the listed datasets can be selected and used to setup the processing scheme. Clicking the Process All button will then apply that processing scheme to all listed datasets. The current implementation generates an output file (in text format) that contains, for each processed file, an index number, the path to the processed FID, and the path to the output dataset. The output file can be loaded by programs such as NMRViewJ to facilitate analysis of the group of spectra.

We are currently implementing access to interleaved datasets, where multiple experiments are collected in a single FID file. These are often used in relaxation analysis and could be processed either by generating multiple 2D files or a single pseudo-3D file.

### Command line processing

The GUI is convenient for interactively setting up processing schemes and visualizing the processed dataset, but all processing can also be done from the command line. Shell scripts are available which take the name of a Python processing script as the single argument. Scripts executed this way will often be used to do repetitive processing on similar datasets or to include any extra Python commands. Processing scripts generated within the GUI can be executed as command line scripts in this way.

Processing via the command line (or direct editing of the GUI based script) currently allows for more detailed control over the processing scheme. One powerful tool that we are currently enhancing is greater access to datasets via Python scripting. For example, it is easy to iterate over all the vectors in a matrix and perform simple math. The vector objects returned during iteration allow various mathematical operations including addition and subtraction. Multiple datasets can be iterated in parallel so a third dataset could, for example, be created by vector addition of two starting datasets. A sample script for this is provided in Listing 4 of Online Resource 2.

### Referencing

The most essential feature of NMR spectra is the chemical shift value, the resonance frequency for the observed nucleus properly scaled and offset relative to a standard reference value. Despite this essential importance, many macromolecular datasets deposited at the BMRB are improperly referenced. This has required significant effort in order to be able to correctly mine the considerable information relating chemical shifts to molecular structure (Aeschbacher et al. 2012; Brown et al. 2015; Wang et al. 2010; Wang and Markley 2009). Furthermore, using both automated and manual techniques for the assignment of spectra require that spectra be referenced properly in order to assess residue type and secondary structure by comparison to database derived chemical shift values. Despite this, it is not unusual for, especially novice, practitioners to wait till they are ready to deposit their data to check the referencing. Accordingly we’ve attempted to make it relatively easy to ensure correct referencing at the start of data processing.

NMRFx Processor is designed to parse the original data files and set up referencing values, but the design of the pulse sequence may make it impossible to directly derive reference values. Accordingly, several Python level commands are available for setting parameters used in processing (sweep width, spectrometer frequency and reference position) in a way to allow for correct data referencing. These commands can take explicit numerical values, but more importantly can use symbolic parameters to extract the correct value from the original data parameter files. For example, the command **sf(‘sfrq’,‘dfrq2’)** could be used to set the spectrometer frequency of the first and second dimension of a dataset from a 2D Agilent experiment to the respective values contained in the ‘sfrq and ‘dfrq2 parameters in the ‘procpar’ file. Any parameters contained in Agilent parameter files (‘procpar’) and Bruker parameter files (‘acqus’, acqu2s’, ‘procs’, ‘proc2s’ etc.) are accessible in this way. Bruker parameter names are specified with the format **parname,n** where n refers to the dimension. So, the sweep widths of a 2D dataset from a Bruker experiment could be specified with **sw(‘SW,1’,‘SW,2’)**. Parameter values can also be extracted with the ‘**p**’ command. This allows the numerical value of the parameter to be used in Python mathematical expressions. For example, **sf(p(‘sfrq’)** **+** **5000.0/1.0e6,‘dfrq’)**, could be used if shifted pulses were used so the effective carrier frequency was 5000.0 Hz from the actual carrier frequency (in MHz). The scheme derived here allows flexible setting of reference information based on raw data parameters. A given processing script can be applied to multiple experiments that may have different actual values (sweep width etc.) without the user needing to specifically enter those values, but allows peculiarities of the pulse sequence (for example, using the third RF transmitter as the source for the second dataset dimension with Agilent experiments).

Proton-detected biomolecular spectra should generally be referenced using an internal shift reference for the proton dimension (DSS) and other nuclei (carbon, nitrogen, etc.) referenced by using indirect referencing ratios relative to the proton carrier frequency and reference value (Wishart et al. 1995). This is made simple in NMRFx Processor by allowing carbon, deuterium, nitrogen and phosphorous frequencies to be referenced by simply indicating the nucleus type. If the proton channel is correctly referenced, and spectrometer frequencies correctly set (as described above), then the correct reference value will be set. For example, the command ref(4.73,‘C’,‘N’) sets the proton reference frequency to 4.73 ppm, and calculates the carbon and nitrogen channels using the appropriate indirect referencing ratios. While using an actual internal standard of DSS is the recommended procedure, it is not uncommon (in part, given concerns about potential DSS interactions with the macromolecule) to set the proton transmitter on the water signal, and use the expected water chemical shift as the reference. This can be done in NMRFx by setting the reference parameter for the proton dimension to ‘h2o’. For example, **ref(‘h2o’, ‘N’)**, would set the proton reference to the typical chemical shift of water at the experimental temperature (derived automatically from the parameter set) and the nitrogen reference shift to the value derived by indirect referencing. Sometimes the user knows the specific chemical shift at a specific spectrometer frequency. This can be added with a format like 0.0@800.3174239.

### Undoing processing operations

Some processing schemes involve applying processing steps to a given dimension, and then after processing another dimension, undoing all the steps. For example, this is common when processing includes linear prediction of the indirect dimensions of 3D (or higher dimensions) with nmrPipe (Delaglio et al. 1995). Processing of a 3D dataset would include processing the second dimension without linear prediction, then processing the third dimension with linear prediction. Then the processing of the second dimension is undone, and it is reprocessed with linear prediction. In this way the number of signals that are present in each of the vectors to be extended with linear prediction is minimized.

NMRFx Processor supports undoing complete dimensions with the UNDODIM command. The UNDODIM command searches for the last Process that has the same dimension as that specified to the command. A new Process object is created whose operation list consists of the inverse of the operations in the original list, in inverse order to which they were originally done. Execution of the Process then effectively undoes the processing done by the original Process object on that dimension. Listing 1 in Online Resource 2 provides an example of this. Currently, the ZF, FT, REAL, PHASE and the various apodization functions can generate an inverse operation. It is essential that parameters of any apodization functions are set so that the apodization window is not near zero at any point as division by a near zero value will result in artifactual large (or infinite) values.

### Skipping dimensions

It’s often useful to process only a subset of the dimensions of the NMR dataset. For example, processing the H^N^–N and H^N^–C dimensions of a 3D HNCO experiment yields two 2D datasets that can be used to check the phasing for the two indirect dimensions. This can be done by including a skip command in the reference section of the processing script with arguments set to 0 for dimensions to include, and 1 for dimensions to skip. For example, **skip(0,1,0)** would process the first and third dimensions, but skip the second. The DIM command and associated operations can be included for the skipped dimensions. This allows the user, for example, to create a full processing script with all dimensions, and simply set the skip command to **skip(0,1,0)** to get the H^N^–N 2D experiment, to **skip(0,0,1)** to get the H^N^–C 2D experiment, and to **skip(0,0,0)** to get the full 3D experiment.

### Non-uniform sampling

A significant advance in NMR data processing over the past decade has been the development and application of a variety of algorithms for processing data collected with non-uniform sampling (NUS) (Kazimierczuk and Orekhov 2015). These NUS experiments allow collection of NMR datasets in significantly shorter time than allowed by conventional uniform sampling schedules and have potential for higher sensitivity relative to uniformly sampled experiments (Palmer et al. 2015). Accordingly it’s essential that new processing programs provide support for these techniques.

A variety of methods for processing non-uniformly sampled data are available. Based on the good results reported with iterative soft thresholding we’ve chosen that method as the first one to implement. Our implementation is similar in principle to a previously described implementation (Hyberts et al. 2012). As the details differ we give a summary of our implementation here. Two features distinguish our approach. First, we do thresholding on only the real values and use a Hilbert transform to regenerate the imaginary values after thresholding. The value of operating on the real values, and avoiding issues with the broad dispersive components, has been recently described (Mayzel et al. 2014; Stern and Hoch 2015). Second, we only replace non-sampled points, leaving sampled points unchanged at the end of the process.

Datasets are first processed with normal methods in the directly detected dimension. Then, for each point in the transformed direct dimension, the corresponding values for the indirectly detected dimensions are extracted into a data object. For a 2D experiment this will be a 1D vector, for 3D experiments a 2D matrix, etc. Then this data object is transformed as follows:

1. Fourier transform the object (multi-dimensional for 2D and higher).
2. Get the real part of the object.
3. Cut intensities above a threshold and copy them to an add buffer. The original values are replaced with the threshold value.
4. Apply a Hilbert transform to the trimmed values (not the add buffer) to regenerate the imaginary values.
5. Inverse Fourier transform the trimmed values.
6. Set all non-sampled values to 0.0.
7. Loop to step 1 (until number of iterations exceeds a specified number).
8. Apply a Hilbert transform to the add buffer.
9. Inverse Fourier transform the add buffer.
10. Copy sampled values from original data into the corresponding position of the add buffer.

At the end of the processing steps above the data object contains original values where values were actually sampled, and calculated values where values were not sampled, and should be a good representation of the data that would exist if full-sampling had been performed. The data object is now stored back into the dataset. Processing of all the indirect dimensions now proceeds as if the dataset were a normally sampled dataset.

The actual implementation is made more efficient by combining the transforms in steps 4 and 5, and steps 8 and 9, above. A typical implementation of the Hilbert transform involves an inverse and then forward Fourier transform of the data. The forward transform can be skipped in this case, as we wish the data to remain in the time domain.

## Results and discussion

NMRFx Processor has been tested on a variety of NMR datasets including one to four dimensional datasets with files generated on Bruker and Agilent spectrometers. Datasets have been processed with iterative soft thresholding for non-uniform datasets. Beginning and advanced users alike have readily adapted to doing their NMR processing via the NMRFx GUI.

### Portability

NMRFx has been designed to be portable across a variety of operating systems. In particular it runs on Mac OS (version 10.7.3 and later), Windows, and varieties of Linux. Writing the low-level code in Java allows us to achieve this portability. Essentially, any operating system that can run Java version 8 or later should support NMRFx. The software is delivered with the Java runtime environment embedded in the application. This frees the end-user from needing to worry about obtaining and installing Java, and whether an installed version will cause conflicts with other software dependent on Java. Portability is demonstrated in Table 2, which provides a list of tested computers and their operating systems along with the time required to process a 3D HNCO dataset with and without linear prediction.

**Table 2:** Examples of execution on multiple computer types

Hardware	Operating system	Number of threads	Compute time (s)	
			HNCO w/o LP	HNCO w/LP
MacBook Pro Intel i7 4 Core	Mac OS 10.10	4	1.7	18.3
MacBook Pro Intel i7 4 Core	Windows 8	4	1.2	16.0
Supermicro Workstation,2 Xeon 6 Core each	Ubuntu 12.04	12	3.6	20.1
Supermicro Workstation,2 Xeon 6 Core each	Windows 7	12	1.9	15.0

These are examples of systems on which NMRFx is tested, but its operation is not limited to the systems listed. Compute times are calculated from processing of a 3D HNCO experiment using a benchmark program (see for example, Listing 3 in Online Resource 2). The number of threads used is chosen to correspond to the number of processing cores

### Parallel processing

Parallel processing will at best result in a speedup equal to n-fold, where n is the number of processors. This level of speedup will not actually occur in practice for a variety of reasons. For example, reading and writing of data from the processor to main memory and ultimately to and from disk drives, may limit performance. Overhead in the code that manages the thread pool may also limit performance. We assessed the performance increase available through parallelization by measuring the elapsed time to process several datasets as a function of the number of threads used.

**Fig. 4:**
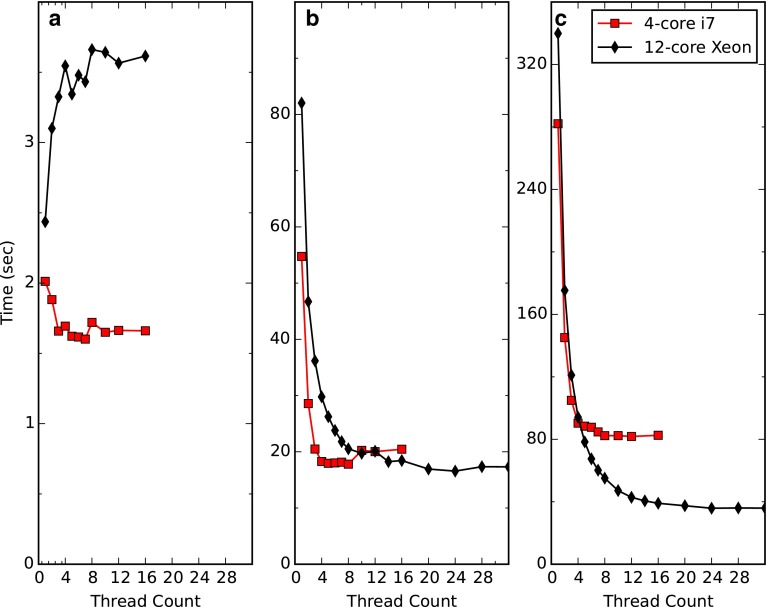
Processing time as a function of the number of processing threads. **a** Three-dimensional HNCO spectrum. **b** Three-dimensional HNCO spectrum processed with linear prediction in the indirect dimensions. **c** Three-dimensional HNCO non-uniformly sampled spectrum processed with iterative soft thresholding. Two computers were used, a MacBook Pro with a 4-core Intel i7 processor running at 2.6 GHz with a solid-state storage drive and a Supermicro Desktop computer with two Intel Xeon 2630 6-core processors running at 2.3 GHz with a conventional disk drive

Figure 4 plots the overall processing time for two datasets, one processed with and without linear prediction, and one with the more time intensive iterative soft thresholding. Performance was measured on a laptop (Apple Macbook Pro) with four cores and a desktop (Supermicro) computer with 12 cores. As can be seen a very significant increase in speed is available for the more computationally intense processing with linear prediction or IST, though as expected it is less than linear in the number of threads. For the NUS dataset, with four threads, the Apple is 3.1 times faster than a single thread and the Supermicro 3.6 times faster. For the optimum thread count as tested the Apple is 3.5 times faster and the Supermicro 9 times faster than when compared to a single thread. Adding threads can actually degrade performance on one computer system (with conventional disk drives) with the less computationally intense processing problem (Fig. 4a). It’s likely that in this case data IO is rate limiting. Further speed increases are likely with careful optimization of the processing code (which we have not yet done). Greater performance increases could be made with distributed processing across multiple computers. The latter, though can require significant effort on the part of users to establish, configure and maintain a processing environment.

### Processing non-uniformly sampled data

NMRFx Processor provides two approaches for processing of non-uniformly sampled data. First, we provide an internal implementation of iterative soft thresholding. Having a good internal implementation provides users with an almost transparent method, relative to standard processing, to process their data. No external programs need be installed and processing can be done on all platforms NMRFx runs on. The user simply needs to include a single additional operation in the processing scheme (this is done by our code that automatically generates processing scripts when vendor parameters indicate that the data has been acquired with non-uniform sampling). But, development of new algorithms for processing non-uniformly sampled data remains an area of active research. New methods are developed faster than we can implement them internally (Qu et al. 2015; Sun et al. 2015), and it’s not always immediately clear which methods are superior and should be chosen for internal implementation. So we also make it possible to include calls to external processing code as part of the overall processing scheme (see Listing 2 in Online Resource 2).

### Educational value

**Fig. 5:**
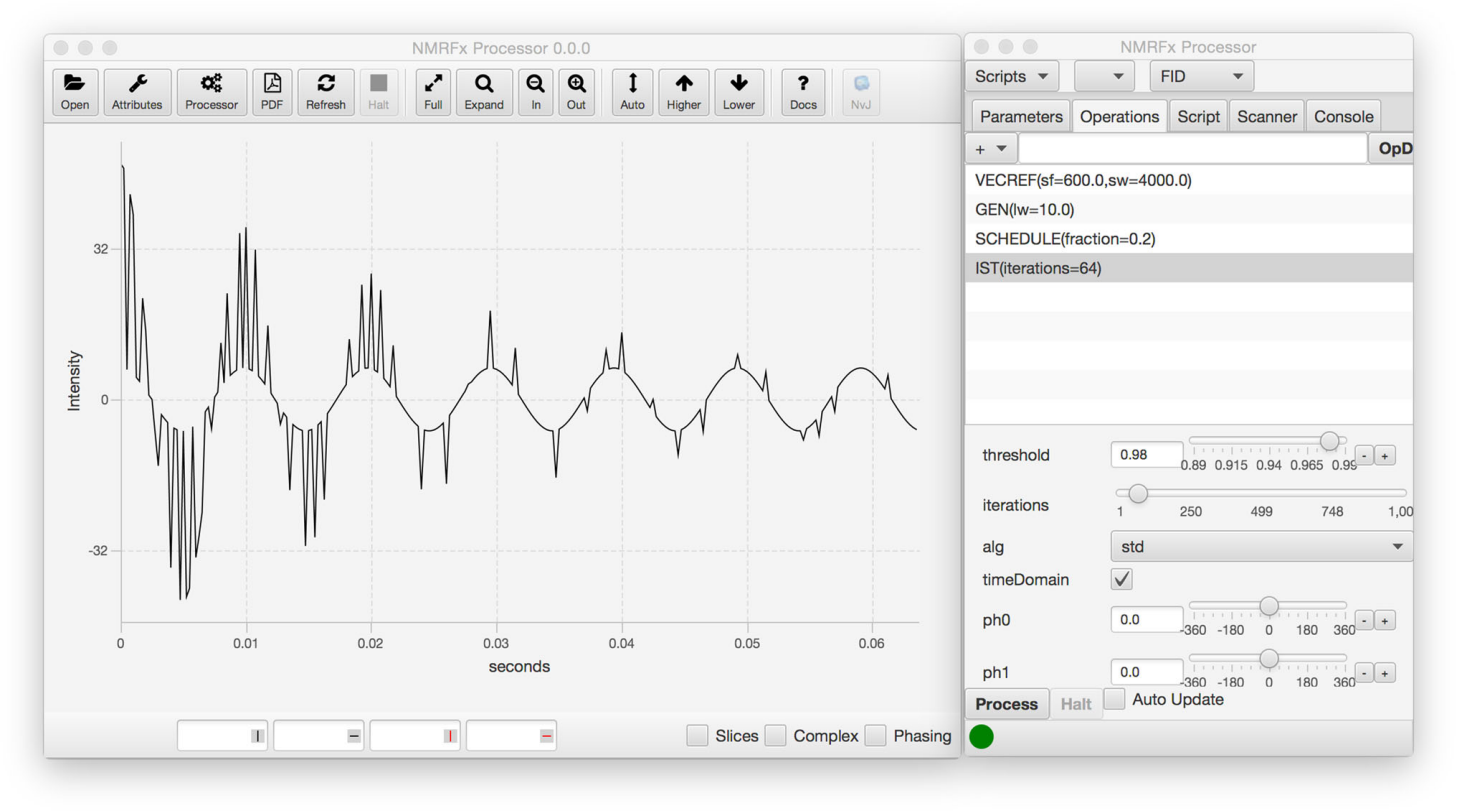
Simulated data with partial IST processing. A simulated FID is generated as if it was recorded on a 600 MHz spectrometer with a sweep width of 4000 Hz and line width of 10 Hz. An operation is included to simulate a non-uniform sampling schedule with 20 % sampling. Finally an IST operation is added to regenerate the unmeasured points. The display is shown with only 64 iterations of the algorithm. Approximately 500 iterations are required to fully reconstruct the data. Students can interactively adjust parameters such as the iteration count to observe the effect on the data

Understanding complex NMR processing can be challenging for the novice user. The NMRFx Processor program has been designed to allow it be used in educational settings. Students can load sample data or simulate FIDs and interactively observe the results of different processing algorithms and parameters. For example, Fig. 5 shows a simulated FID with fractional non-uniform sampling and its processing with IST. Interactively changing parameters such as the number of iterations of the iterative soft thresholding algorithm can be used to observe how missing data points are replaced with values generated with the algorithm.

## Conclusions

NMRFx Processor has been written to be a powerful, yet easy to use, application that is applicable to processing a wide variety of NMR datasets. To date, it’s been tested on a variety of datasets during development and is being used by a growing number of external laboratories. We expect, given the almost infinite variety of NMR experiments, that there will certainly be experiments requiring further development and optimization to properly process them, but the fundamental toolset available within the application should make this relatively straightforward.

The application takes advantage of the multi-core processors available in almost all modern laptop and desktop computers. We’ve done relatively little so far to optimize performance and while performance is already quite good there should be significant room for improvement. In particular, we expect that many datasets can be loaded completely in memory. This should minimize the time used for input/output and allow increased benefits from the parallel execution on multi-core computers. We are also investigating running code in parallel on compute clusters and we are beginning to develop versions of the code that can harness the power of Graphics Processing Units (GPUs).

The rich variety of built-in operations, the existing and expanding interactions with the Python scripting tools, and the modern GUI combine to make NMRFx a powerful and user-friendly new tool for NMR spectroscopists. We anticipate that it will be used for both routine processing needs and form a toolset upon which new processing techniques can be developed.

## Electronic supplementary material

Below is the link to the electronic supplementary material.

Supplementary material 1 (PDF 128 kb)

Supplementary material 2 (TXT 4 kb)
