# Supplementary material for: NMRFx Processor: a cross-platform NMR data processing program
Source: J Biomol NMR. 2016 Jul 25;65:205–16. doi: 10.1007/s10858-016-0049-6 (PMC4983292; doi:10.1007/s10858-016-0049-6)
Supplement: Supplementary file 1 — Supplementary material 1 (PDF 128 kb) [file 10858_2016_49_MOESM1_ESM.pdf]

ADD Add value to the vector at all points between first and last, where value can either be an integer (real) or a complex number written as (1.0 + 3j).

- **value** The value to add to each data point.
  - **default** 0j
  - **optional** True
- **first** The first point of the vector to add to.
  - **default** 0
  - **min** 0
  - **max** size - 1
  - **optional** True
- **last** The last point of the vector to add to.
  - **default** -1
  - **min** -1
  - **max** size - 1
  - **optional** True

AUTOPHASE Auto Phase shift.

- **firstOrder** Do first order phase correction.
  - **default** False
  - **optional** True
- **maxMode** Autophase by maximizing positive signal.
  - **default** False
  - **optional** True
- **winSize** Size of each half of window used in doing CWTD. Full window is 2 x this value.
  - **default** 2
  - **min** 1
  - **max** 32
  - **optional** True
- **ratio** Ratio relative to noise used in determining if region is signal or baseline.
  - **default** 25.0
  - **min** 1.0
  - **max** 100.0
  - **optional** True

AUTOREGIONS Baseline correction using a polynomial fit.

- **mode** Specify the mode for auto identifying baseline regions.
  - **default** sdev
  - **optional** True
- **winSize** Size of window used in searching for baseline regions;
  - **default** 16
  - **min** 4
  - **max** 256
  - **optional** True
- **minBase** Baseline regions must be at least this big;
  - **default** 12
  - **min** 4
  - **max** 256
  - **optional** True

- **ratio** Ratio relative to noise used in determining if region is signal or baseline.
  - **default** 10.0
  - **min** 1.0
  - **max** 100.0
  - **optional** True

BCPOLY Baseline correction using a polynomial fit.

- **order** Order of the polynomial used in fit;
  - **default** 2
  - **min** 1
  - **max** 8
  - **optional** True
- **winSize** Size of window used in searching for baseline regions;
  - **default** 16
  - **min** 4
  - **max** 256
  - **optional** True

BCSINE Baseline correction using a sine curve.

- **regions** Specify the points of the vector to perform baseline correction on.
  - **default** None
  - **optional** True
- **type** Specify the units for the region values.
  - **default** pts
  - **optional** True
- **invert** Specify the boundary of peaks instead of the baseline.
  - **default** False
  - **optional** True
- **order** Order of the polynomial used in fit;
  - **default** 1
  - **min** 1
  - **max** 8
  - **optional** True
- **winSize** Size of window used in searching for baseline regions;
  - **default** 16
  - **min** 4
  - **max** 256
  - **optional** True
- **ratio** Ratio relative to noise used in determining if region is signal or baseline.
  - **default** 0.0
  - **min** 1.0
  - **max** 100.0
  - **optional** True

BCWHIT Baseline correction using a smoother.

- **lamb** Parameter controlling how close the fit to the baseline should be
  - **default** 5000
  - **min** 1000.0

    - **max** 20000.0
    - **optional** True
- **order** Order of the polynomial used in fit;
    - **default** 1
    - **min** 1
    - **max** 2
    - **optional** True
- **baseline** If true, return the calculated baseline, rather than the corrected vector
    - **default** False
    - **optional** True

BUCKET The vector is bucketed by adding adjacent data points. The vector size after this operation will be equal to the specified number of buckets. The original vector size must be a multiple of the number of buckets. Each resulting data point will represent the sum of winSize data points where winSize is equal to size/nBuckets

- **buckets** Number of buckets to place data points into. Vector size must be a multiple of this number.
    - **default** 256
    - **min** 0
    - **max** size
    - **optional** True

BZ Zero Bruker DSP baseline and associated algorithms: *sim, ph, dspph, chop*.

- **alg** Algorithm to correct Bruker DSP artifact.
    - **default** ph
    - **optional** True
- **phase** Phase adjust (sim, ph only).
    - **default** 0.0
    - **min** -180
    - **max** 180
    - **optional** True
- **scale** Scale factor (sim only).
    - **default** 1.0
    - **min** -1
    - **max** 3
    - **optional** True

COMB combine inVec and outVec with a list of coefficients

- **coef** How to combine data rows with different phases.
    - **default** None
    - **optional** True

CSHIFT Circular shift of the data points in the vector by the specified amount.

- **shift** Amount of points to shift the vector by.
    - **default** 0
    - **min** -2048
    - **max** 2048
    - **optional** True

CWTD Continuous Wavelet Transform Derivative.

- **winSize** Size of the window.
  - **default** 32
  - **min** 1
  - **max** 1024
  - **optional** True

DC Shifts the spectrum so edges are centered. DC Offset.

- **fraction** The fraction of points from the beginning and end of a spectrum that will be used to create the offset.
  - **default** 0.05
  - **min** 0
  - **max** .33
  - **optional** True

DCFID Correct DC offset of FID real and imaginary channels

- **fraction** Fraction of end of FID to average to calculate offset
  - **default** 0.06
  - **min** 0.01
  - **max** 0.25
  - **optional** True

DX Numerical Derivative.

EA Do echo-anti echo combination

ESMOOTH Envelope smoothing.

- **winSize** Size of the window
  - **default** 256
  - **optional** True
- **lambd** Parameter controlling how close the fit to the baseline should be
  - **default** 5000
  - **min** 1000.0
  - **max** 50000.0
  - **optional** True
- **order** Parameter controlling the order of the baseline fit
  - **default** 2
  - **min** 1
  - **max** 2
  - **optional** True
- **baseline** If true, return the calculated baseline, rather than the corrected vector
  - **default** False
  - **optional** True

EXP Exponential Calculation of a Vector. Each point is updated with the exponential value of the point .

EXPD Exponential Decay Apodization.

- **lb** Line broadening factor.
  - **default** 1.0
  - **min** 0.0
  - **max** 20.0
  - **optional** True
- **fPoint** First point multiplication.
  - **default** 1.0
  - **min** 0.5
  - **max** 1.0
  - **optional** True

EXTRACT Extract a specified range of points.

- **start** Start point of region to extract
  - **default** 0
  - **min** 0
  - **max** size-1
  - **optional** True
- **end** End point of region to extract
  - **default** 0
  - **min** 0
  - **max** size-1
  - **optional** True
- **mode** Extract a named region (left,right,all,middle) instead of using start and end points
  - **default** left
  - **optional** True

FDSS Frequency Domain Solvent Suppression.

- **center** Position of frequency to suppress. Default is in fractional units with zero at center..
  - **default** 0.0f
  - **min** -0.5
  - **max** 0.5
  - **optional** True
- **start** The beginning of the peak.
  - **default** 0.005f
  - **min** 0.00
  - **max** 0.010
  - **optional** True
- **end** The end of the peak.
  - **default** 0.015f
  - **min** 0.00
  - **max** 0.02
  - **optional** True
- **autoCenter** Find the largest peak in spectrum and center on that.
  - **default** False
  - **optional** True

FILTER Generic filter, type is *notch* or *lowpass*.

- **type** Filter type.

- **default** notch
- **optional** True
- **offset** Frequency offset in fraction of sw.
  - **default** 0
  - **min** -0.5
  - **max** 0.5
  - **optional** True
- **width** Notch width in fraction of sw (notch only).
  - **default** 0.05
  - **min** 0.01
  - **max** 0.09
  - **optional** True
- **factor** Decimation factor (lowpass only).
  - **default** 4
  - **min** 3
  - **max** 20
  - **optional** True
- **groupFactor** Filter sharpness.
  - **default** 8
  - **min** 4
  - **max** 40
  - **optional** True
- **mode** Filter type.
  - **default** zero
  - **optional** True

FT Fourier Transform.

- **negateImag** Negate imaginary values before the FT
  - **default** False
  - **optional** True
- **negatePairs** Negate alternate complex real/imaginary values before the FT
  - **default** False
  - **optional** True
- **auto** Determine negatePairs from FID parameters
  - **default** False
  - **optional** True

GAPSMOOTH Solvent suppression by removing signal and filling the gap with a smoothing function.

- **center** Center point of the solvent peak.
  - **default** -1
  - **optional** True
- **start** Beginning point of the solvent peak.
  - **default** -1
  - **optional** True
- **end** End point of the solvent peak.
  - **default** -1
  - **optional** True
- **autoCenter** Find largest peak in spectrum and set that as center
  - **default** False

- **optional** True

GEN Generate a simulated signal and add it to the vector.

- **freq** Frequency in Hz.
  - **default** 100.0
  - **min** -500
  - **max** 500.0
  - **optional** True
- **lw** Linewidth in Hz.
  - **default** 1.0
  - **min** 0
  - **max** 10.0
  - **optional** True
- **amp** Amplitude of signal.
  - **default** 50.0
  - **min** 0
  - **max** 100.0
  - **optional** True
- **phase** Phase of signal in degrees.
  - **default** 0.0
  - **min** -180
  - **max** 180.0
  - **optional** True

GF Lorentz-to-Gauss.

- **gf** gf: Gaussian broadening
  - **default** 1.0
  - **min** 0.0
  - **max** 20.0
  - **optional** True
- **gfs** gfs: Gaussian center
  - **default** 1.0
  - **min** 0.0
  - **max** 1.0
  - **optional** True
- **fPoint** fpoint: First point multiplier
  - **default** 1.0
  - **min** 0.0
  - **max** 1.0
  - **optional** True

GM Lorentz-to-Gauss.

- **g1** g1: Exponential line narrowing
  - **default** 1.0
  - **min** 0.0
  - **max** 20.0
  - **optional** True
- **g2** g2: Gaussian broadening

  - **default** 1.0
  - **min** 0.0
  - **max** 20.0
  - **optional** True
- **g3** g3: Gaussian center
  - **default** 0.0
  - **min** 0.0
  - **max** 1.0
  - **optional** True
- **fPoint** fpoint: First point multiplier
  - **default** 1.0
  - **min** 0.0
  - **max** 1.0
  - **optional** True

GMB Gauss Broaden Window.

- **gb** Gaussian Broadening Coefficient.
  - **default** 0.0
  - **min** 0.0
  - **max** 1.0
  - **optional** True
- **lb** Line broadening.
  - **default** 0.0
  - **min** -20.0
  - **max** 20.0
  - **optional** True
- **fPoint** Factor multiplied with the first point.
  - **default** 1.0
  - **min** 0.0
  - **max** 1.0
  - **optional** True

HFT Hilbert Transform

IFT Inverse Fourier Transform

IMAG Set the real values equal to the imaginary values and discard the rest.

INTEGRATE Set the signal equal to its integral. int : first First point of integration region int : last Last point of integration region

IST Iterative Soft Threshold.

- **threshold** Values above this threshold (multiplied times largest peak) are transfered to IST add buffer.
  - **default** 0.98
  - **min** 0.89
  - **max** 0.99
  - **optional** True
- **iterations** Number of iterations to perform.
  - **default** 500

    - **min** 1
    - **max** 1000
    - **optional** True
- **alg** Name of algorithm to use.
    - **default** std
    - **optional** True
- **timeDomain** Is the end result of the operation in time domain
    - **default** True
    - **optional** True
- **ph0** Apply this zero order phase correction to data before IST.
    - **default** None
    - **min** -360.0
    - **max** 360.0
    - **optional** True
- **ph1** Apply this first order phase correction to data before IST.
    - **default** None
    - **min** -360.0
    - **max** 360.0
    - **optional** True
- **adjustThreshold** Adjust threshold during IST calculation
    - **default** False
    - **optional** True
- **zeroFill** Zero fill vector during IST calculation
    - **default** True
    - **optional** True

ISTMATRIX Iterative Soft Threshold for 2D Matrix.

- **threshold** Values above this threshold (multiplied times largest peak) are transfered to IST add buffer.
    - **default** 0.9
    - **min** 0.1
    - **max** 1.0
    - **optional** True
- **iterations** Number of iterations to perform.
    - **default** 500
    - **min** 1
    - **max** 1000
    - **optional** True
- **alg** Name of algorithm to use.
    - **default** std
    - **optional** True
- **phase** Array of phase values, 2 per indirect dimension.
    - **default** None
    - **optional** True

LP Extend the vector using Linear Prediction. Forward or backward linear prediction can be done. If both are specified then both are done and coefficients averaged (forward-backward LP).

- **fitStart** First point used in fit. Defaults to 0 or 1 (depending on forward/backward mode) if 0;
    - **default** 0
    - **min** 0

    - **max** size-1
    - **optional** True
- **fitEnd** Last point used in fit. Defaults to size-1 if 0.
    - **default** 0
    - **min** 0
    - **max** size-1
    - **optional** True
- **predictStart** Position of first predicted point. Defaults to size if 0.
    - **default** 0
    - **min** 0
    - **max** size-1
    - **optional** True
- **predictEnd** Position of last predicted point. Defaults to 2*size-1 if 0.
    - **default** 0
    - **min** 0
    - **max** size*2-1
    - **optional** True
- **npred** Number of points to predict, only used if predictEnd is 0.
    - **default** 0
    - **min** 0
    - **max** size*2-1
    - **optional** True
- **ncoef** Number of coefficients. Defaults to size/2 if 0.
    - **default** 0
    - **min** 0
    - **max** size-1
    - **optional** True
- **threshold** Threshold of singular values used in keeping coefficients. Check this??
    - **default** 5
    - **min** 4
    - **max** 10
    - **optional** True
- **backward** Do backwards linear prediction.
    - **default** True
    - **optional** True
- **forward** Do forwards linear prediction.
    - **default** True
    - **optional** True
- **mirror** Do mirror image linear prediction.
    - **default** None
    - **optional** True

LPR Replace starting points of the vector using Linear Prediction. Forward or backward linear prediction can be done. If both are specified then both are done and coefficients averaged (forward-backward LP).

- **fitStart** First point used in fit. Defaults to 0 if 0;
    - **default** 0
    - **min** 1
    - **max** size-1
    - **optional** True
- **fitEnd** Last point used in fit. Defaults to size-1 if 0.

- **default** 0
- **min** 0
- **max** size-1
- **optional** True

- **predictStart** Position of first predicted point. Defaults to 0 if < 0.
  - **default** 0
  - **min** 0
  - **max** size/4
  - **optional** True
- **predictEnd** Position of last predicted point. Defaults to 0 if 0.
  - **default** 0
  - **min** 0
  - **max** size/4
  - **optional** True
- **npred** Number of points to predict, only used if predictEnd is 0.
  - **default** 0
  - **min** 0
  - **max** size*2-1
  - **optional** True
- **ncoef** Number of coefficients. Defaults to size/2 if 0.
  - **default** 0
  - **min** 0
  - **max** size-1
  - **optional** True
- **threshold** Threshold of singular values used in keeping coefficients. Value used is 10^-threshold
  - **default** 5
  - **min** 3
  - **max** 10
  - **optional** True
- **backward** Do backwards linear prediction.
  - **default** True
  - **optional** True
- **forward** Do forwards linear prediction.
  - **default** True
  - **optional** True

MAG Magnitude Calculation of a Vector. Each point is updated with its Complex magnitude.

MEASURE Measures regions in spectrum.

- **key** Prefix to key used to store measure values in a map (dictionary). Key will have vector row appended.
  - **default** measures_
  - **optional** True
- **map** Map in which to store results. If not specified (or = None) the default map will be used. Get the default map with "getMeasureMap()"
  - **default** None
  - **optional** True

MULT Multiply the points in a vector by a Real or Complex number.

- **value** Number to multiply the points by.
  - **default** (1+0j)
  - **optional** True
- **first** Points starting from this will be multiplied by value. Default is 0.
  - **default** 0
  - **min** 0
  - **max** size - 1
  - **optional** True
- **last** Last point to multiply the data by. Default is the end of the vector.
  - **default** -1
  - **min** -1
  - **max** size - 1
  - **optional** True

ONES Set all points in a vector to 1.0

PHASE Phase shift.

- **ph0** Zero order phase value
  - **default** 0.0
  - **min** -360.0
  - **max** 360.0
  - **optional** True
- **ph1** First order phase value
  - **default** 0.0
  - **min** -360.0
  - **max** 360.0
  - **optional** True
- **dimag** Discard imaginary values
  - **default** False
  - **optional** True

POWER Power Calculation of a Vector. Each point is updated with its power value.

PRINT Print vector.

RAND Set all points in a vector to a uniformly distributed random number between 0.0 and 1.0.

RANDN Add a Gaussian to a vector.

- **mean** Mean of the Gaussian.
  - **default** 0.0
  - **min** 0.0
  - **max** 100.0
  - **optional** True
- **stdev** Standard deviation of the Gaussian.
  - **default** 1.0
  - **min** 0.1
  - **max** 100.0
  - **optional** True
- **seed** Seed for the RNG.

- **default** 0
    - **min** 0
    - **optional** True

RANGE Sets the values in the vector from first to last inclusive to either the specified value (which can be real or complex (written as 1.0 + 3j) or Double Min or Double Max.

- **value** Vector will have this value from the 'first' to 'last' elements
    - **default** 0j
    - **optional** True
- **first** The first point of the vector to set.
    - **default** 0
    - **min** 0
    - **max** size-1
    - **optional** True
- **last** The last point of the vector to set.
    - **default** -1
    - **min** -1
    - **max** size-1
    - **optional** True
- **max** Set the value to Double.MAX (instead of min or value). If True, overrides value.
    - **default** False
    - **optional** True
- **min** Set the value to Double.MIN (instead of max or value). If True, overrides value.
    - **default** False
    - **optional** True

REAL Make the vector real, discarding the imaginary part

REGIONS Baseline correction using a polynomial fit.

- **regions** Specify the points of the vector to perform baseline correction on.
    - **default** None
    - **optional** True
- **type** Specify the units for the region values.
    - **default** frac
    - **optional** True
- **signal** Specify the boundary of peaks instead of the baseline.
    - **default** False
    - **optional** True

REVERSE Reverse points in a vector

RFT Real fourier transform

- **inverse** True if inverse RFT, False if forward RFT.
    - **default** False
    - **optional** True

SB Sine Bell Apodization

- **offset** Offset of sine window.

    - **default** 0.5
    - **min** 0.0
    - **max** 0.5
    - **optional** True
- **end** End value of sine window argument.
    - **default** 1.0
    - **min** 0.5
    - **max** 1.0
    - **optional** True
- **power** Exponential power.
    - **default** 2.0
    - **min** 1.0
    - **max** 2.0
    - **optional** True
- **c** First point multiplier.
    - **default** 1.0
    - **min** 0.5
    - **max** 1.0
    - **optional** True
- **apodSize** Size of apodization window. Default 0f 0 uses entire FID.
    - **default** 0
    - **min** 0
    - **max** size
    - **optional** True

SCHEDULE Sets a sample schedule for a 1D vector and zeros points not on schedule. Used for testing IST.

- **fraction** The fraction of points that are collected.
    - **default** 0.05
    - **min** 0.05
    - **max** 1.0
    - **optional** True
- **endOnly** If true, only zero values at end of vector
    - **default** False
    - **optional** True

SCRIPT Execute a Python script as an Operation. Current vector is available as object named "vec".

- **script** The script that will be run on each Vec at the stage in the processing queue.
    - **default**
    - **optional** True
- **initialScript** Any initial declarations that will be executed on initialization.
    - **default**
    - **optional** True
- **encapsulate** Whether the interpreter should persist between evaluations or be reinitialized for each evaluation.
    - **default** False
    - **optional** True

SHIFT Left or right shift of the data points in the vector by the specified amount.

- **shift** Amount of points to shift the vector by.
  - **default** 1
  - **min** -16
  - **max** 16
  - **optional** True

SIGN Change sign of values

- **mode** What elements of vector to change .
  - **default** i
  - **optional** True

SQRT Sqrt Calculation of a Vector. Each point is updated with its square root.

TDCOMB combine complex inVec and outVec time domain vectors using a list of coefficients

- **dim** Indirect dimension of dataset to combine vectors in. Use 2 for 2D, 2 or 3 for 3D, etc.
  - **default** 2
  - **optional** True
- **coef** How to combine data rows with different phases.
  - **default** None
  - **optional** True

TDPOLY Time Domain Polynomial.

- **order** Order of the polynomial.
  - **default** 4
  - **min** 1
  - **max** 10
  - **optional** True
- **winSize** Size of the window
  - **default** 32
  - **min** 1
  - **max** size-1
  - **optional** True
- **start** First point
  - **default** 0
  - **min** 0
  - **max** size-1
  - **optional** True

TDSS Time domain solvent suppression.

- **winSize** Window size of moving average filter (+/- this value).
  - **default** 31
  - **min** 1
  - **max** 128
  - **optional** True
- **nPasses** Number of passes of filter. Three is optimal.
  - **default** 3
  - **min** 1

    - **max** 3
    - **optional** True
- **shift** Position of frequency to suppress. Default is in fractional units with zero at center..
    - **default** 0.0f
    - **min** -0.5
    - **max** 0.5
    - **optional** True

TM Trapezoid Multiply.

- **pt1** First point to multiply.
    - **default** 0
    - **min** 0
    - **max** size-1
    - **optional** True
- **pt2** Last point to multiply.
    - **default** -1
    - **min** -1
    - **max** size-1
    - **optional** True

TRI Triangle Window

- **pt1** Middle point of the triangle.
    - **default** 0
    - **min** 0
    - **max** size-1
    - **optional** True
- **lHeight** Height of the left side.
    - **default** 1.0
    - **min** 0.0
    - **max** 1.0
    - **optional** True
- **rHeight** Height of the right side.
    - **default** 0.0
    - **min** 0.0
    - **max** 1.0
    - **optional** True

VECREF Sets size, spectrometer frequency and sweep width of vector. Used for simulated FIDs for testing and demonstration.

- **size** Size of vector specified as a power of 2.
    - **default** 8
    - **min** 3
    - **max** 16
    - **optional** True
- **sf** Spectrometer frequency (in MHz).
    - **default** 500.0
    - **min** 0.0
    - **max** 1200.0

    - **optional** True
- **sw** Sweep width of spectrum (in Hz).
    - **default** 5000.0
    - **min** 0.0
    - **max** 10000.0
    - **optional** True

WRITE Write vector to dataset (normally done automatically).

ZEROS Zeros a vector.

ZF Zero Fill. factor is the 'factor' power of 2 that the vector size is increased to, so if the vector has 513 elements and factor = 1, it will increase to 1024, the next power of 2, but if factor = 2, it will increase to 2048, which is two powers of two greater. A size can be specified instead of a factor which will be the exact number of points the vector will have, and the increased elements will all be zero.

- **factor** Number of powers of 2 to zero fill to.
    - **default** 1
    - **min** -1
    - **max** 4
    - **optional** True
- **size** Size after zero filling. If -1 (default), calculate from factor value.
    - **default** -1
    - **min** -1
    - **max** 65536
    - **optional** True
- **pad** Increase size by this amount. If -1 (default) use size or factor value.
    - **default** -1
    - **min** -1
    - **max** 128
    - **optional** True
